# A narrative review on the environmental impact of medicines: from water analysis and *in vivo* studies to prescribing appropriateness, based on European and Italian legal frameworks

**DOI:** 10.3389/fdsfr.2025.1681648

**Published:** 2025-12-18

**Authors:** Irene Cristini, Elisabetta Poluzzi, Federica Soardo, Salvatore Crisafulli, Stefano Polesello, Ursula Kirchmayer, Marco Milani, Francesca Russo, Michele Nicoletti, Giovanna Scroccaro, Paola Deambrosis, Shima Tavakolian Haghighi, Jacopo Grisotto, Ugo Moretti, Giovanna Paolone, Gianluca Trifirò

**Affiliations:** 1 Department of Diagnostics and Public Health, University of Verona, Verona, Italy; 2 Department of Medical and Surgical Sciences, University of Bologna, Bologna, Italy; 3 National Research Council, Water Research Institute, CNR-IRSA, Brugherio, Monza and Brianza, Italy; 4 Department of Epidemiology, Lazio Regional Health Service, Rome, Italy; 5 Regional Directorate of Prevention, Food Safety, Veterinary Public Health - Regione del Veneto, Venice, Italy; 6 Regional Directorate of Pharmaceutical, Prosthetic, Medical Device - Regione del Veneto, Venice, Italy

**Keywords:** one health, environmental pharmacoepidemiology, environmental risk assessment, sustainable prescribing, pharmaceutical pollution

## Abstract

The environmental impact of medicines has become a growing concern within the One Health framework, which emphasizes the interconnection of human, animal, and environmental health. This narrative review, based on discussions from a 2025 Italian multistakeholder roundtable, explores current strategies for assessing and reducing such impact, including water monitoring, *in vivo* studies using animal models, prescribing appropriateness, and the relevant European and Italian legal frameworks. The review highlights the crucial role of different stakeholders, including pharmaceutical industries, regulatory agencies, health authorities, researchers, and citizens, in implementing preventive measures to reduce the environmental impact of medicines. It underscores the urgency of integrating environmental sustainability into clinical practice and health policies, encouraging green pharmaceutical innovation, environmentally conscious prescribing, and coordinated governance, in line with the One Health principles. Future research to assess and minimize the environmental footprint of medicines should prioritize the development of standardized metrics for environmental impact, evaluate the long-term efficacy of preventive and mitigation strategies, included those on access to treatments and sustainability of production, and strengthen interdisciplinary collaboration to translate One Health into actionable policies and practices.

## One Health approach

Over the last decades, the importance of considering the interplay between human, animal, and environmental health has grown significantly, and the COVID-19 pandemic has ultimately underscored the urgency of adopting this principle. The World Health Organization states that “One Health is an integrated, unifying approach that aims to sustainably balance and optimize the health of people, animals, and ecosystems. One Health can help to address the full spectrum of disease control–from prevention to detection, preparedness, response, and management–and contribute to global health security” (World Health Organization, 2023a).

**FIGURE 1:**
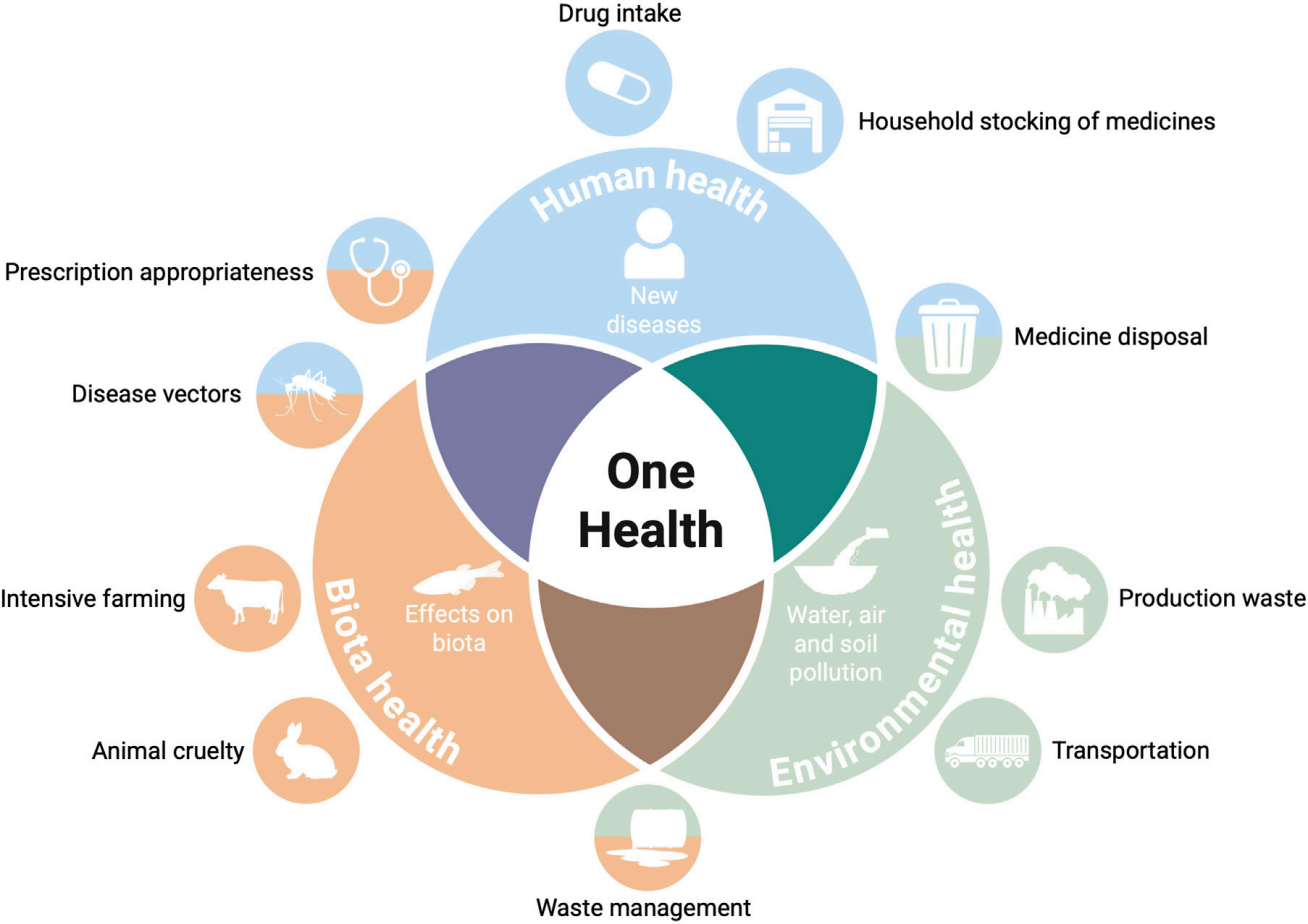
An overview on the impact of medicines on One Health. Created in BioRender. Paolone, G. (2025) https://BioRender.com/3i5vmt8.

Medicines play an important role in both human and veterinary health, and attention to their environmental impact has increased in the last decades. One example at international level is the 28th United Nation Climate Change Conference (COP28) Declaration on Climate and Health (World Health Organization, 2023b), which addresses the environmental impact of medicines by focusing on decarbonizing the healthcare sector and its supply chains, encouraging signatories to curb emissions and waste through greener procurement standards. In recent years, the interdisciplinary field of environmental pharmacoepidemiology has been promoting research in four key areas: environmental pollution caused by medicines throughout their life cycle (from production to waste disposal); the relationship between drug use and climate change (e.g., carbon footprint of inhalers or medical gases); the environmental impact of clinical research (e.g., limiting non necessary clinical studies); and the interactions between the effects of drug use and environmental exposures on human health (e.g., additive risks). An overview of the impact of medicines on One Health is reported in Figure 1.

The main challenges in mitigating this phenomenon include the limited and disjointed availability of regulation, which leads to fragmented competencies and responsibilities among different stakeholders, the high heterogeneity across regions in organization, data access, and data quality, the absence of a common terminology among researchers from different disciplines, and the fact that citizens are often regarded merely as external parties (Forward. Recenti progressi, 2024).

An integrated approach requires an overarching organizational structure enabling interaction among equal partners, along with common and well-defined rules, roles, and responsibilities. It should incorporate One Health as a cross-cutting discipline in education and training across fields, while also routinely integrating citizen science. Furthermore, this approach should be systematically incorporated into regulatory frameworks and policy agendas to ensure sustainability, accountability, and long-term impact. Such integration implies embedding environmental assessments throughout the medicinal product lifecycle, establishing interoperable surveillance systems linking human, animal, and environmental data, and creating intersectoral governance structures to coordinate action.

To promote the One Health approach in drug sciences, a fundamental step is to bring stakeholders together to thoroughly discuss and develop strategies for minimizing the environmental impact of medicines at multiple levels, while prioritizing the need to assure the best available treatments for patients. These include environmental monitoring campaigns, the evaluation of behavioral and biological effects, prescribing practices, and policy or regulatory interventions. Additionally, it is crucial to foster research at the intersection of environmental sciences and pharmacoepidemiology.

This narrative review is based on an Italian multistakeholder round table entitled “Impact of medicines on environment: which perspectives?” that was held at the University of Verona in February 2025. The event brought together nine experts, including representatives from the Italian national association of pharmaceutical companies (*Farmindustria*), academic experts in eco-pharmacovigilance and drug utilization, researchers in environmental pollution, and regional officers from Veneto, particularly representatives from the Department of Prevention, Food Safety and Veterinary Affairs and the Department of Pharmaceutical, Prosthetic and Medical Devices. The round table utilized a structured Question-and-Answer format, moderated by GT and GS, to stimulate a multi-perspective discussion among the involved experts. The resulting insights not only highlighted key challenges and priorities for eco-pharmacovigilance in Italy but also informed potential avenues for evidence-based decision-making regarding the sustainable management of pharmaceuticals. Key outcomes emerging from the discussion included enhanced patient awareness, the fostering of responsible pharmaceutical disposal methods, and the promotion of prescribing appropriateness to comprehensively incorporate environmental considerations. Therefore, the aim of this review is to provide an in-depth overview of the various strategies employed to assess the environmental impact of medicines, ranging from water analysis to *in vivo* studies and the analysis of drug consumption in the context of European and Italian legal frameworks. By bringing together perspectives from different stakeholders, it highlights the need for a collaborative engagement to reduce the environmental burden of pharmaceuticals within a One Health framework.

## How to assess the environmental risk of medicines from water monitoring and ecotoxicological data?

Environmental risk assessment (ERA) of pharmaceuticals is an essential process aimed at safeguarding ecosystems from potential harm caused by medicines. Since 2006, the European Medicines Agency has recognized that ERA should be included in the dossier for the marketing authorization of human medicinal products (HMP) (Committee for Medicinal Products for Human Use, 2024). The recently revised version of this guideline (Committee for Medicinal Products for Human Use, 2024) introduced several new advances meeting the demands of the European Union (EU) Commission 2023 proposal to reform pharmaceutical legislation, which also seeks to strengthen ERAs (European Parliament and Council, 2023). Significant changes are the introduction of ERA for generics and new rules for refusing marketing authorization due to an incomplete or insufficient ERA as requested by Article 15 of the Regulation proposal (European Parliament and Council, 2023). The scopes of ERA are broadened to include more specific hazards like endocrine disruption and antimicrobial resistance, as well as secondary poisoning, to strengthen a persistent, bioaccumulative and toxic (PBT) assessment. A tiered approach is introduced where the mandatory Phase I aims to establish whether the risk assessment process for a specific active substance should proceed to a detailed fate-and-effects assessment with further experimental studies. The Phase I relies separately on a PBT assessment of each active substance present in the HMP, and a risk assessment based on the calculation of risk ratios (RR) (RR = predicted environmental concentration (PEC)/predicted no-effect concentration (PNEC)), with a fixed threshold value for predicted environmental concentration in surface water (PEC_SW_) of 0.01 µg/L. This fixed threshold represents a regulatory screening criterion used in the Phase I ERA to determine whether further evaluation is required. Specifically, if the calculated PEC_SW_ is ≤0.01 µg/L, the compound is considered unlikely to pose an environmental risk; if it exceeds this value, a higher-tier (Phase II) assessment is performed using compound-specific ecotoxicological data (Committee for Medicinal Products for Human Use, 2024). Following this ERA guideline, only potential environmental risks are assessed, since this is the only possibility in the case of pre-sale authorization of HMPs.

In Italy the Italian Medicines Agency (AIFA) introduced an environmental risk assessment of 114 active pharmaceutical ingredients (API) in the 2023 national report on medicines use in Italy (The Medicines Utilisation Monitoring Centre, 2024). API were selected based on three criteria, i.e., highest use in routine care, highest environmental toxicity and inclusion/application in at least one version of the European Watch List (WL). The environmental risk has been assessed at national level, by geographical areas and regions by calculating the classical RR. Considering a null removal in wastewater treatment plants and the number of Italian residents in 2023 in the different macro-areas and regions, PECs have been estimated from the consumption data for all reimbursement classes (Nation Health System-covered and private purchase). To estimate the environmental risk, PNEC values from the NORMAN ecotoxicology database, whenever available, have been extracted (NORMAN-Network, 2009).

Alternative approaches for potential risk assessment have been developed by scoring different physicochemical characteristics and (eco) toxicological endpoints. The potential risks can be derived by the weighted sum of all the scores (Dürig et al., 2019) or, alternatively, as the product between the global score of the physicochemical characteristics (linked to the likelihood of the occurrence of the adverse event) and the global score of the hazard parameters (representing the severity of the event), according to the classical risk equation: Risk=Likelihood x Severity (Polesello et al., 2024).

It is important to emphasize that these approaches aim to highlight the potential impact of a molecule on the aquatic environment, regardless of the availability of data concerning their water concentrations, because they are based on physicochemical and ecotoxicological data that can be retrieved from official and shared databases. International institutions provide open-source databases, such as the United States (US) Environmental Protection Agency’s Ecotoxicology Knowledgebase (ECOTOX) (EPA, 2025) and PubChem by the US National Institutes of Health (PubChem, 2025). In Europe the classification of the environmental hazards and risks of pharmaceuticals can be retrieved from a public Swedish website, Janusinfo (Janusinfo, 2025), based on the Pharmaceutical Specialties in Sweden (*Farmaceutiska Specialiteter i Sverige,* FASS) database provided by the Swedish pharmaceutical industries. PNECs for most of the active ingredients and some metabolites have been estimated from experimental and modelled data by the NORMAN Network, an EU association of public and private environmental laboratories and institutions, and they have been made publicly available in the NORMAN ecotoxicology database (NORMAN-Network, 2009). A collection of PNECs of human pharmaceuticals and metabolite has also been published in 2023 (Giunchi and Poluzzi, 2023). In contrast, the availability of actual occurrence data and measured environmental concentrations is critical to get a reliable RR, which is fundamental for identifying the APIs with the greatest impact on the environment, to prioritize the efforts to reduce their environmental risks. With this purpose, national initiatives have been carried out modeling the PECs of APIs on the basis of the sales data, considering the worst-case conditions of no degradation in the wastewater treatment plants (Agerstrand et al., 2009; Giunchi et al., 2025a). However, this assumption overlooks the key role of persistence in the exposure pathway, which affects the target organisms, humans, and the ecosystems on which they depend on (Davenport et al., 2022). Modeling PECs of APIs solely on sales data presents several additional limitations. First, this approach assumes complete consumption of the purchased API, whereas treatment adherence is often less than 100% (The Medicines Utilisation Monitoring Centre, 2024). Second, interindividual variability in drug metabolism and excretion further affects the accuracy of such a model (Welch et al., 2022).

The need for actual environmental data concerning hundreds of APIs led to the development of monitoring protocols for wide screening of water samples based on innovative techniques such as the high-resolution mass spectrometry hyphenated with chromatographic separations. The screening protocols could be used as a tool to select the target pharmaceuticals to be monitored and quantitatively determined in a tiered approach (Cappelli et al., 2022). The application of this monitoring protocol in a routine monitoring scheme or in a full national monitoring program is hampered by the complexity to manage hundreds of compounds that, in many cases, are not stable and generate multiple metabolites. Furthermore, an accurate mass spectrometric determination requires the use of stable isotope-labeled internal standards for each analyte, thus significantly increasing the overall cost when analyzing a broad panel of APIs and their metabolites. For these reasons, it is preferable to select a limited number of compounds that are representative of the specific study objectives. For example, to investigate the environmental impact of veterinary drugs, antibiotics such as sulfamethoxazole, lincomycin, and clindamycin can be chosen. Pharmaceutical monitoring should target wastewater treatment plant influents and effluents, hospital discharges, river reaches upstream/downstream of outfalls, drinking-water reservoirs, groundwater wells, and co-located sediments and resident biota (European Commission, 2009). Sampling should combine 24-h flow-proportional composites with targeted campaigns during hydrologic extremes (i.e., storm pulses, low-flow). Seasonal trends in drug use (e.g., influenza peaks and non-steroidal anti-inflammatory drugs) should also be considered, with at least monthly sampling for trend detection. To ensure relevance and representativeness of the readings, validated methods (e.g., chromatographic–mass-spectrometric methods) should be applied, and analyses should include matched upstream controls and report both concentrations and flow-normalized loads. Furthermore, accumulation in soil should also be assessed by sludge and leachate monitoring, and bioaccumulation should be evaluated by pairing water with biota analyses (European Commission, 2009).

The availability of measured monitoring data of API in aquatic matrices is also central in the mechanism of watch list monitoring developed under the Water Framework Directive (European Parliament and Council, 2000), the EU regulation for the protection of waters by chemical pollution. APIs, selected as priority substance candidates, by using the modelled PEC or partially available data, are monitored around the EU in a fixed set of sampling sites for a mandatory time range to improve the spatial and temporal reliability of measured environmental concentrations used in the RR evaluation. WLs compounds undergo a biennial review (Gomez et al., 2025). Based on this evaluation, substances are either promoted to Priority Substance status and consequently removed from the WL, or they are retained on the WL for an additional period, not to exceed 4 years, until sufficient data is generated (Gomez et al., 2022). For instance, diclofenac was removed after adequate monitoring data supported its promotion to Priority Substance under the Environmental Quality Standards Directive (European Parliament and Council, 2008). The WL approach led to the proposal of introducing for the first time some pharmaceuticals (i.e., 17 alpha-ethinylestradiol, 17 beta-estradiol, estrone, azithromycin, carbamazepine, clarithromycin, diclofenac, erythromycin, and ibuprofen) in the priority substances list (European Parliament and Council, 2022). The quality status of the water bodies is classified according to the compliance of monitoring data of the priority substances with their environmental quality standards (EQS). One weakness of this regulatory architecture is that deriving reliable EQS requires extensive experimental ecotoxicological data, with a special emphasis on sub-lethal endpoints and matrices other than waters (Backhaus, 2023; Whitehouse, 2001). For this reason, it is recommended to make publicly available the institutional and research-based databases, along with the assessment document of the ERA, which is compiled by the applicant and submitted to the regulatory authority (Oelkers, 2020). As an ultimate goal, this approach would help collect PNECs, based on the most up-to-date information, which is essential for RR assessment for single compounds (Giunchi and Poluzzi, 2023).

However, the RR measurement for single compounds has the limitation of overlooking the combined toxic effects of a mixture of low doses of many biologically active molecules. It is very unlikely to evaluate in detail the synergistic vs. antagonistic effects of a mixture of thousands of possible APIs, but simpler models based on the additive hypothesis, e.g., using the toxicity equivalency concept, have been developed and tested on real samples (Escher et al., 2008; Palumbo et al., 2022). From a monitoring perspective, effect-based methods are available for detecting specific receptor-mediated effects, such as estrogenic activity. *In vitro* bioassays are highly efficient and precise, utilizing cell lines that are genetically engineered to constitutively express specific receptors (Neale et al., 2025). For instance, the VM7Luc4E2 and VM7Luc4ER bioassays express the estrogenic receptor, making them well-suited for evaluating the agonistic or antagonistic effects of environmental contaminants on the estrogenic signaling pathway (Frieberg et al., 2023). However, effect-based bioassays can lack sensitivity for low-level contaminants and, while they reveal overall biological activity without identifying specific chemicals, they struggle to quantify individual contributions within mixtures. A potential approach to address these limitations involves integrating multiple *in vitro* and *in vivo* bioanalytical assays to comprehensively assess biological responses and elucidate the effects of contaminant mixtures, as focusing solely on a singular chemical or *in vitro/in vivo* monitoring offers only a limited and insufficient insight (Brack et al., 2019). These methods can detect the integrated effect of all active chemicals in a sample extract, including both known and unknown chemicals, and can account for mixture effects. For some of these methods, linked to well-characterized receptor responses, threshold or trigger values can be derived to carry out an RR measurement (Neale et al., 2023).

For an integrated view of the whole toxicity profile of a mixture, the simplest regulatory solution is currently the introduction of a Mixture Assessment Factor (MAF) as a pragmatic tool to account for potential mixture risks during the risk and safety assessment of individual chemicals (Backhaus, 2023). The MAF is derived from exposure modeling and empirical data on mixtures, designed to adjust single-substance risk metrics to better represent mixture hazards. This factor is particularly considered for substances produced in large quantities, and different MAFs may be tailored according to pharmacodynamic profiles or chemical classes. MAFs provide a simple and practical way to assess chemical mixtures but are limited by data gaps and modeling challenges. They should be used as a cautious tool within a stepwise risk assessment approach (Backhaus, 2023). Nevertheless, this approach has been suggested for mixtures of industrial chemicals in the EU’s Chemicals Strategy for Sustainability, and it is currently under discussion for inclusion in the Registration, Evaluation, Authorization and Restriction of Chemicals (REACH) revision.

## How does the presence of medicines in aquatic environments affect animal behavior?

Once the presence of medicines in the water has been demonstrated, it is of paramount importance to evaluate their neurobehavioral impact on animals.

More specifically, the increasing presence of pharmaceutical pollutants in aquatic environments has raised concerns over their effects on the behavior and health of aquatic organisms. Pharmaceuticals discharged into aquatic environments can reach soils through pathways like wastewater irrigation, sewage sludge use, and surface runoff (Pérez-Lucas and Navarro, 2024). Once in the soil, they are absorbed by microorganisms, plants, and land-dwelling invertebrates (Mosharaf et al., 2024). These compounds tend to accumulate in organisms such as earthworms, plants, and riparian spiders, resulting in bioaccumulation and possible biomagnification across terrestrial food webs (Säfholm et al., 2014). Studies have also shown that emerging aquatic insects can transfer pharmaceuticals from water systems into terrestrial ecosystems (Previšić et al., 2021). Moreover, pharmaceutical pollutants may cause shifts in soil microbial activity, disrupt soil fungi, and threaten terrestrial biodiversity, with risks of antibiotic resistance and entry into the human food chain via food products and crops (Lunghi et al., 2025). Acute toxicity of pharmaceuticals in aquatic environments can disrupt reproduction, development, and physiological processes in aquatic species. Evidence shows that drugs like diclofenac and ibuprofen, commonly detected in river and bay waters across the globe at concentrations from tens to thousands of ng/L, induce cardiac abnormalities, impair gamete maturation, and reduce fertility in fishes and invertebrates (Parolini, 2020). These chemicals also provoke oxidative stress, DNA damage, and endocrine disruption, affecting tissue biomarkers and organ function (Świacka et al., 2021). Pharmaceuticals and their transformation products can accumulate in animal tissues, and acute exposures may quickly elicit effects like embryonic deformities, metabolic stress, and reproductive impairment even in the absence of visible behavioral changes (Garg et al., 2023; Hejna et al., 2022; Sangion and Gramatica, 2016). Furthermore, pharmaceuticals in aquatic environments can negatively impact biota across all levels, including microorganisms, fungi, and plants, by disrupting growth, metabolism, and reproduction. They inhibit microbial community functions, affect fungal diversity, and suppress algal and plant productivity, leading to altered nutrient cycling and ecosystem balance even at low concentrations (Ortúzar et al., 2022).

As a well-established model species in ecotoxicological and biomedical research, adult zebrafish (*Danio rerio*) exhibits a wide range of complex and quantifiable behaviors, making them ideal for assessing sublethal impacts of pharmaceutical exposure (Ortúzar et al., 2022).

To investigate the neurobehavioral effects of pharmaceutical contaminants, researchers strive to replicate realistic environmental conditions in laboratory settings. This includes maintaining accurate pharmaceutical concentrations through either direct dosing or using real wastewater samples to simulate authentic exposure scenarios (Szychlinska and Marino Gammazza, 2025). Environmental parameters such as pH, temperature, and light cycles must be carefully controlled during testing, as described in the Organization for Economic Co-operation and Development (OECD) Test No. 230 (OECD, 2009). These factors impact the bioavailability and toxicity of pharmaceuticals by affecting chemical stability and fish physiology. Maintaining consistent conditions minimizes variability, ensuring that observed effects can be reliably attributed to the test substance rather than environmental fluctuations, thus enhancing the accuracy and reproducibility of the assay results. Behavioral assays like the startle response, general locomotion tests, and anxiety-like assessments such as the novel tank or light-dark tests are routinely exploited to evaluate stress and neurotoxicity in both larval and adult zebrafish (Gendelev et al., 2024; Grisotto et al., 2025; Ochenkowska et al., 2022). Pharmaceutical exposure can profoundly influence locomotor activity in adult zebrafish by altering neurotransmitter systems and acting on neurotransmitter receptors and transporters. A clear example is the effect of fluoxetine, a selective serotonin reuptake inhibitor (SSRI) frequently detected in aquatic environments. Exposure to 0.54 or 54 µg/L of fluoxetine demonstrated that zebrafish displayed a significant reduction in their exploratory behavior and swimming activity compared with their matched controls (Vera-Chang et al., 2019). The lower exposure concentration directly reflects environmentally relevant levels found in effluents and surface waters impacted by wastewater (up to 929 ng/L (Martínez Bueno et al., 2007)), meaning the observed behavioral and endocrine effects in zebrafish are indeed ecologically relevant for aquatic systems globally (Martínez Bueno et al., 2007). Fluoxetine also impairs shoaling behavior by increasing the distance between individuals in a group, likely due to altered serotonergic signaling (Cueto-Escobedo et al., 2021). The disruption in cohesion and the increase in solitary exploration may reduce the effectiveness of group-based defense strategies and synchronized behavior. Furthermore, exposure to citalopram results in increased surface-level swimming in novel tank tests, suggesting that SSRIs alter motor function and spatial behavior, as reported by Gendelev et al. (2024) and Szychlinska and Marino Gammazza (2025). Among others, venlafaxine is a commonly detected compound in surface waters. The observed levels of venlafaxine in the aquatic environment range from 50 ng/L (Schlüsener et al., 2015) to 2,190 ng/L (Schultz and Furlong, 2008) in European and North American waterbodies, respectively. Exposure to this antidepressant with concentrations of 1, 10, and 100 μg/L for 20 days has shown to reduce the frequency of mating behavior in adult zebrafish, raising concerns about its potential to disrupt reproductive processes and population dynamics in aquatic organisms (Tang et al., 2022). These behavioral alterations could impair key survival mechanisms such as foraging, predator avoidance, and reproduction.

In addition, according to Sackerman et al., exposure to anxiolytic agents like diazepam reduced defensive behaviors and promoted increased exploration in novel environments (Sackerman et al., 2010). Diazepam also altered the natural zebrafish preference for dark surroundings, shifting the preference to illuminated areas for the females and exacerbating the preference for dark enclosures for males (Grisotto et al., 2024b). Along with this, Brand et al. demonstrated that Atlantic salmon (*Salmo salar*) exposed to another common aquatic pollutant, the benzodiazepine clobazam, prior to release exhibited altered migratory behavior, with a greater proportion of exposed individuals reaching the sea compared with controls. Subsequent analyses confirmed the presence of clobazam in the brains of these fish, highlighting the ecological relevance of pharmaceutical contamination in aquatic environments (Brand et al., 2025). In contrast, anxiogenic compounds like yohimbine heighten stress responses in zebrafish, triggering behaviors such as freezing or bottom-dwelling. These effects are tied to alterations in neurotransmitter systems, particularly those involving GABAergic and adrenergic signaling pathways (Petersen et al., 2022). Another common contaminant, atenolol, also demonstrated induction of anxiety-like behavior in zebrafish. As observed in the novel tank test, 1.0 and 10 µg/L atenolol caused a significant increase in the latency to enter the top area, reduction in the number of transitions to the top area, and time spent in the top area. These symptoms could be due to atenolol-mediated interference in serotonergic and cholinergic neurotransmission, and brain-derived neurotrophic factor expression (Adedara et al., 2024).

Pharmaceuticals also affect zebrafish social behaviors, which are essential for group survival and reproductive success. Exposure to major representative pharmaceuticals like acetaminophen, carbamazepine, metformin, and trimethoprim at environmentally relevant concentrations individually as well as in a mixture, impacts the social preferences of male zebrafish, together with alterations in light-dark preference and tank exploration (Dhakshinamoorthy et al., 2025). Similarly, amoxicillin and clavulanic acid induce social alterations in both sexes, as seen in the social preference test, and also hamper locomotion and anxiety-like behavior in the novel tank test (Grisotto et al., 2024a). Furthermore, venlafaxine exposure has been linked to increased aggression, influencing social hierarchies and mating interactions (Gendelev et al., 2024).

The behavioral effects of pharmaceutical exposure in zebrafish extend beyond individual health, posing broader ecological consequences. Decreased locomotor function may hinder escape responses and efficient foraging, while altered anxiety responses can shift habitat use and increase vulnerability to predation. Disruptions in social behaviors compromise reproduction and social structure, threatening population stability (Gendelev et al., 2024; Ochenkowska et al., 2022; Szychlinska and Marino Gammazza, 2025).

Therefore, understanding the mechanisms by which pharmaceutical pollutants affect animal behavior offers valuable insight into their neuromodulatory potential. Behavior, as an indicator of central nervous system functioning, may reveal underlying biochemical disturbances through these alterations. This behavioral-molecular approach, as an effective model for connecting ecotoxicology and human neuropharmacology and for aiding in the prediction of the effects of low-dose, persistent exposures on brain function, enhances environmental risk assessment and supports the development of more protective public health policies. Therefore, the integration of molecular and behavioral endpoints should be further explored to enhance the robustness of ERA frameworks, which currently rely predominantly on bioactivity and toxicity-based assays. Incorporating these complementary endpoints can offer a more comprehensive understanding of sublethal and mechanism-specific effects of contaminants. In particular, the use of advanced model organisms, such as adult zebrafish, enables the investigation of complex physiological and behavioral responses, thereby improving the ecological relevance, accuracy, and predictive power of risk evaluations.

## How to incorporate the environmental impact into the prescribing appropriateness assessment?

Use of medicines in real-world settings is guided by the clinical evidence on their benefit-risk profiles, which is incorporated in treatment guidelines issued by national and international scientific societies. Clinical guidelines are the undisputed reference for prescribers to choose medications on the basis of the aforementioned evidence, together with their applicability in clinical practice (i.e., including assessment of economic and social sustainability).

With the growing awareness of the environmental impact of medicines, efforts of regulators and scientists toward including this dimension in the risk-benefit profile of diagnostic and therapeutic choices have been increasing (Senerth et al., 2025). Particularly, a discussion has risen on incorporating environmental considerations into the health technology assessment (HTA) (Greenwood Dufour et al., 2022; Toolan et al., 2023). In this regard, in Toolan et al. (2023), identified four potential approaches to help HTA agencies integrate environmental assessments into healthcare decision-making. These include information conduit (i.e., republishing public domain or HTA-submitted data), parallel evaluation (i.e., presenting environmental data separately from health economic analyses), integrated evaluation (i.e., combining clinical, financial, and environmental data in a single quantitative analysis) and environment-focused evaluation (i.e., analyzing technologies with environmental benefits but no expected health outcome improvement).

Therefore, the environmental risk should be added to the overall risk profile associated with each medication. As a consequence, among medicines that are considered to be comparable in terms of efficacy and safety, those with a lower environmental impact should be prioritized. Since an agreement on quantification of the environmental impact for each single medication is still ongoing (Guirado-Fuentes et al., 2023; Pinho-Gomes et al., 2022), the current approach to specific cohorts of patients should follow a decision-making flowchart that outlines actions to prevent or minimize the environmental impact of medicines (especially on surface water). The above-mentioned flowchart is based on the following questions:

1. Is the use of this medicine clinically appropriate for that specific cohort of patients (or this single patient)?
2. What is its expected environmental impact?
3. Are there therapeutic alternatives (comparable in terms of efficacy and safety) with lower environmental impacts (either medicinal products or non-pharmacological approaches)?
4. Are there behavioral recommendations for patients to limit this impact?

Undoubtedly, the first recommendation is for prescribers and patients to limit medication overuse/misuse. Antibiotics are the most suitable example in this regard, considering both their well-known abuse and their impact on the environmental microbiome, with the obvious consequence of an increase in resistant strains of bacteria and effects on public health. For instance, despite the implementation of the WHO AWaRe Antibiotic Book (World Health Organization, 2022) as national prescribing guidance, Italy remains the seventh-highest consumer of antibiotics in Europe (The Medicines Utilisation Monitoring Centre, 2025). This high consumption is particularly driven by an excessive use of ‘Watch’ and ‘Reserve’ antimicrobial agents, contributing to Italy being among the countries with the highest percentage resistance rates for several critical pathogen-antibiotic combinations (The Medicines Utilisation Monitoring Centre, 2025).

Medication review and deprescribing are also becoming important strategies to limit medication overuse and misuse, especially among older patients with complex multimorbidity (Carollo et al., 2024; Crisafulli et al., 2022). These patients often maintain long-term therapeutic regimens without subsequent pharmaceutical reconciliation, despite potential changes in their underlying medical conditions. As a consequence, not only does their risk of adverse drug reactions increase (Toh et al., 2023), but the environmental burden imposed by these drugs, considering their entire life cycle, from manufacturing to excretion, is clinically and ecologically unjustified (Department of Health and Social Care, 2021). Conservative prescribing and regular medication reviews are essential to improve the clinical outcomes of patients and the environmental impact of prescribing, but when the prescriptions are essential, supplying the exact number of doses for a single course to the patient would improve the environmental damage caused by drugs and improve adherence to the therapeutic regimen (Hänninen et al., 2023). Additional opportunities include packaging with compact formats, avoiding empty spaces, as pharmaceutical packaging often accounts for a substantial portion of a drug’s total ecological footprint (Bassani et al., 2022), and reducing packaging sizes particularly for new drug treatments and starter packs (Paut Kusturica et al., 2022).

The second recommendation is to consider all appropriate pharmacological alternatives and, when the benefit–risk profiles are comparable, prefer the option that maximizes sustainability, including costs, patient accessibility, and environmental impact. Since agents with similar clinical efficacy often belong to the same pharmacological class and may exhibit similar environmental behavior, the substitution of one same-class compound for another is not necessarily expected to reduce environmental burden. Therefore, the de-prioritization of a drug on environmental grounds should be justified by robust, compound-specific evidence (e.g., higher PEC/PNEC, poor removal in wastewater treatment, persistence or bioaccumulation), to avoid regrettable substitutions. The limited availability of standardized and comparable data concerning the environmental risks posed by pharmaceuticals currently hinders comprehensive comparative analyses. To address this gap, the EU and the European Federation of Pharmaceutical Industries and Associations (EFPIA) are jointly sponsoring PREMIER (PREMIER, 2020a). This project is aimed at developing a novel information and assessment system for the identification and management of the environmental risks of drugs. Furthermore, this new framework could be used to prioritize and screen older APIs that have never been through an ERA, ultimately establishing a basis for environmental data comparisons (PREMIER, 2020b). Although Italy does not yet maintain a national database analogous to Sweden’s Janusinfo (Janusinfo, 2025), AIFA has recently mapped environmental risks using national utilization data and surface-water exposure models (Giunchi et al., 2025a). This analysis identified high-priority compounds for environmental risk management, providing a foundation for evidence-based stewardship decisions (The Medicines Utilisation Monitoring Centre, 2024). This approach can be implemented at regional and local levels and aligned with international examples, such as the Stockholm Wise List (The Stockholm Drug and Therapeutics Committee, 2015), where, when the pharmaceuticals are equivalent in terms of medical efficacy, safety and pharmaceutical effectiveness, their environmental impact and price are also taken into account. To integrate environmental information into clinical decision-making, technical data must be presented in a format that is comprehensive and relevant to non-specialists. Therefore, translation into clear qualitative categories (e.g., low, moderate, or high environmental risk), along with a guidance on interpretation and clinical implications, as implemented in the Janusinfo database (Janusinfo, 2025), can enhance usability in clinical practice.

Moreover, when clinically appropriate, therapeutic strategies should encompass non-pharmacological approaches, such as dietary modifications and smoking cessation, or psychological treatments, notably cognitive behavioral therapy for mental health and nature-based or outdoor activities to promote holistic wellbeing (Twohig-Bennett and Jones, 2018). At the patient level, behavioral recommendations should be pragmatic and harmonized with national prescribing practices. These include dispensing and prescribing medication quantities consistent with the intended duration of treatment, reinforcing adherence to avoid incomplete therapeutic courses, and promoting the return of unused or expired medicines to authorized pharmacy collection systems rather than their disposal via household waste or sinks. The heightened public concern regarding the health consequences of climate change has fostered widespread support for the different measures implemented to reduce the environmental impact of pharmaceuticals and healthcare systems. This support is further demonstrated by the citizenship’s willingness to change their behavior, for instance by returning the unused medicines or empty inhalers to the pharmacy for reuse or disposal and accepting the exact amount of medication prescribed without being given excessive amounts (Cameron et al., 2021; Pegg et al., 2024; Sadreghaemy et al., 2025). In Italy, citizens are encouraged to dispose of their unused or expired medicinal products at the authorized municipal collection points, and then these are managed by Assinde, the national distributed collection system for pharmaceutical waste (Assinde, 2003).

When the most clinically appropriate medication for major disorders is suspected to be highly dangerous for the environment, its use must be guaranteed, but actions to minimize its environmental impact should be taken. Source reduction interventions aimed at preventing the hazardous API from reaching the general wastewater system include: dedicated collection of high-risk excreta (e.g., using urine bags following the use of X-ray contrasting agents (Niederste-Hollenberg et al., 2018)); protocol improvements to minimize contamination (e.g., refining surface cleaning and wiping hands before washing (European Society of Oncology Pharmacy, 2018; Presidente della Repubblica, 2003)); and supplying patients with the exact number of doses required for a single treatment course.

## What is the role of the pharmaceutical industry in developing preventive strategies for medicine-induced environmental effects?

All stakeholders play a crucial role in minimizing the environmental impact of medicines. In detail, the pharmaceutical industry is committed to ensuring the environmental safety of medicinal products across their life cycle through the implementation of the ERA (Committee for Medicinal Products for Human Use, 2024; Giunchi et al., 2025b), which is part of the marketing authorization application in the EU. The environmental risk posed is the result of the intrinsic hazards of the APIs, their use and exposure in the environment.

Concurrently, pharmaceutical companies have been investing in greener manufacturing processes to limit API emissions into wastewater and have been adopting green chemistry principles to develop molecules with lower persistence and ecotoxicity. In this regard, many initiatives can be mentioned, such as the European Eco-Pharma Stewardship (European Federation of Pharmaceutical Industries and Associations, 2015), focused on improving scientific understanding, finding new ways to detect the trace amounts of pharmaceuticals in the environment and understanding their impact, and the Anti-Microbial Resistance (AMR) Industry Alliance (AMR Industry Alliance, 2017), which has developed a framework for assessing and promoting environmentally responsible discharge targets for antibiotic manufacturing. Furthermore, the EU has funded five Horizon Europe (Europa, 2021) research–industry collaborative projects under the Innovative Health Initiative (IHI) (Innovative Health Initiative, 2021). Collectively, these projects form the Green Pharma Cluster (Enviromed, 2022; ETERNAL Project, 2025; Mechanochemistry, 2022; SusPharma, 2022; TransPharm, 2022), which aims to increase the sustainability of pharmaceutical products and exploit research synergies to boost the impact of innovations. Other important IHI–supported projects include PREMIER (PREMIER, 2020a), which seeks to establish a comprehensive framework for assessing and characterizing the environmental risks associated with APIs, and PHARMECO (Pharmeco, 2024), aimed at promoting sustainable industrial transition and developing scalable, eco-friendly manufacturing optimizations and innovations.

At the EU policy level, the 2024 revision of the Urban Wastewater Treatment Directive (UWWTD) (European Parliament and Council, 2024) imposed obligations for pharmaceutical companies that complement the above-mentioned measures. It requires quaternary advanced treatment for micropollutants at wastewater treatment plants and establishes an Extended Producer Responsibility (EPR) scheme for producers of human medicinal and cosmetic products. Based on this directive, by 31 December 2028, Member States must ensure producers collectively cover at least 80% of the full costs of quaternary treatment, the costs of monitoring micropollutants and of gathering and verification of market data, with contributions modulated by quantity and hazardousness of substances placed on the market (European Parliament and Council, 2024). This makes the UWWTD the most significant recent EU measure on pharmaceuticals in the environment because, unlike risk assessment and voluntary source-control programs, it directly assigns a share of downstream removal costs to producers, creating incentives for greener-by-design APIs and formulations that reduce persistence (and thus future EPR liabilities). This directive has led to a still-ongoing debate, as industry associations argue that concentrating costs on medicines could affect access and affordability for patients (European Federation of Pharmaceutical Industries and Associations, 2024; Medicines for Europe, 2025).

At the national level, the Italian national association of pharmaceutical companies (*Farmindustria*), together with the supply chains of both private and public pharmacies (*Federfarma* and *Assofarm*) and of pharma distributors (*Associazione Distributori Farmaceutici and Federfarma Servizi*), established Assinde (Assinde, 2003), a centralized system to guarantee the correct disposal of expired medicines and prevent them from ending up in the environment. Since 2003, Assinde has ensured the correct disposal of over 1,000 tons of pharmaceutical waste every year, through a pharmaceutical reverse distribution and waste management system. Pharmacies across the national territory provide citizens with designated expired medication collection points. Assinde subsequently manages the reverse logistics for the unsalable pharmaceutical products, ensuring their final corrective disposal by high-temperature incineration (Assinde, 2003; Presidente della Repubblica, 2003). Moreover, the Italian national association of over-the-counter medicines (*Federfarma Assosalute*) launched an informational campaign on the correct disposal of drugs, aimed at increasing public awareness (Federfarma Assosalute, 2023). The initiative provided detailed guidance on how different types of pharmaceutical packaging (e.g., blisters, vials, syringes, transdermal patches) should be appropriately discarded and underscored that expired medicines must be returned to authorized pharmacy collection systems for safe disposal. The pharmaceutical industry has increasingly acknowledged its responsibility to reduce its environmental impact throughout the life cycle of medicinal products (Pharmaceutical Supply Chain Initiatives, 2023). While the sector is progressively integrating sustainability into research, manufacturing, and supply chain processes, it emphasizes the need for science-based, risk-sensitive and “technologically neutral” approaches to avoid unintended consequences for medicine availability and patient needs. Importantly, the transition toward environmentally sustainable practices requires a careful balance between ecological objectives and the sector’s competitiveness, which remains essential to sustaining investment and innovation in both healthcare and environmental sustainability (European Federation of Pharmaceutical Industries and Associations, 2024). In this context, the Pharmaceutical Environmental Group (PEG Hub.org, 2023a) has established, in partnership with the Sustainable Markets Initiative (Sustainable Markets.org, 2023), the Pharmaceutical Life-Cycle Assessment Consortium (PEG Hub.org, 2023b) to develop harmonized, science-based methodologies for the Life-Cycle Assessment (LCA) of medicinal products, which is a process aimed at evaluating the environmental impacts of a drug from its “cradle to grave”, including its development, manufacturing, packaging, transportation, use, and end-of-life (Satta et al., 2024). This initiative reflects the industry’s collective commitment to transparent, comparable, and evidence-driven approaches aimed at reducing environmental impacts, while safeguarding patient access and promoting innovation.

## What is the role of regional and national health authorities in developing preventive strategies on medicine-induced environmental effects?

In parallel, public health authorities play a key role in mitigating the environmental impact of pharmaceuticals. Their growing engagement has been driven not only by public health imperatives but also by the progressive integration of health system responsibilities into environmental dimensions.

The European regulatory framework laid the foundation for this integration (European Parliament and Council, 2000; 2006) and introduced the concept of chemical pollutants in aquatic environments. These directives established minimum requirements, while allowing Member States to enact stricter regulations. Subsequent acts (European Commission, 2022; European Parliament and Council, 2019) further emphasized the importance of surveillance and control of pharmaceutical residues in the environment.

In Italy, these provisions were transposed in 2006 with the “*Testo Unico Ambientale*” (Gazzetta Ufficiale della Repubblica Italiana, 2006). Since then, the role of health institutions in environmental matters has progressively grown, in parallel with the consolidation of the One Health approach, which recognizes the interconnection between human, animal, and environmental health.

At the national level, Italy has formalized this approach by establishing the National Prevention System for Health Risks from Environmental and Climate Factors (SNPS), a coordination structure enabling dialogue between human health, veterinary, and environmental sectors (Gazzetta Ufficiale della Repubblica Italiana, 2021).

At the regional level, these national strategies are adapted and extended. The Veneto Region has implemented a dedicated Regional Prevention System (SRPS) to overcome sectoral fragmentation and promote integrated governance (Regione del Veneto, 2025). Furthermore, the Veneto Region is developing a unified data infrastructure, the Veneto Data Platform, to interconnect environmental, veterinary, and health databases. This tool facilitates both emergency response and strategic planning in a scientifically grounded, One Health evidence-based framework.

Other instruments have also evolved at the national level. For example, the National Plan for Combating Antimicrobial Resistance (PNCAR 2022–2025) includes explicit references to environmental issues, encouraging monitoring of resistant pathogens and pharmaceuticals through wastewater-based surveillance (Italian Ministry of Health, 2022). The PNCAR aims to provide strategic guidance and operational directions to address the emergency of AMR, adopting a One Health approach with three main intervention areas: i. integrated surveillance and monitoring of antibiotic resistance, antibiotic use, healthcare-associated infections, and environmental monitoring; ii. prevention of healthcare-associated infections in hospital and community settings, and of infectious diseases and zoonoses; iii. appropriate use of antibiotics in both human and veterinary contexts, and proper management and disposal of antibiotics and contaminated materials (Italian Ministry of Health, 2022).

An innovative implementation of this approach is the reconversion of the Environmental Wastewater Surveillance in Italy (*Sorveglianza Ambientale Reflue in Italia,* SARI) (Italian Institute of Health, 2025), which was originally designed to detect SARS-CoV-2 in wastewater during the COVID-19 pandemic and is now being repurposed for multi-pathogen surveillance.

Moreover, in the Veneto region, several initiatives (Regione del Veneto, 2024) are dedicated to educating both the public and healthcare professionals on the appropriate use of antimicrobials and the risks associated with their misuse, in both human and veterinary contexts. These efforts, endorsed by the Veneto Region and organized by the local health authorities, include, for instance, public awareness meetings (e.g., “Antimicrobial resistance (*L’antibiotico resistenza*)” and “The silent pandemic of antimicrobial resistance (*La pandemia silenziosa dell’antibiotico-resistenza*)”), the distribution of informative material at medical clinics and pharmacies, and targeted workshops with clinicians and healthcare professionals (Regione del Veneto, 2024).

These multi-level governance efforts demonstrate the essential role of public health authorities in transforming EU environmental principles and national and regional One Health policies into actionable and coordinated strategies to reduce the pharmaceutical burden on ecosystems and safeguard population health.

However, many challenges still remain and continuous collaboration among industry, regulators, and academia is essential to address environmental impacts while responding to patient needs and ensuring access to medicines.

## Future directions

Many stakeholders are involved in defining and applying strategies to minimize the impact of medicines on the aquatic environment: from pharmaceutical industries (limiting residues of production and designing greener pharmaceuticals), to healthcare systems and clinical researchers (incorporating the environmental impact of medicines into their rational use), and eco-toxicologists and environmental agencies (estimating toxicity of medicinal products in terms of both threshold of toxicity of each single molecule and assessing actual residues in surface waters, upgrading water treatment plants). Each stakeholder is asked to strengthen interactions with all other “environmental players”, including regulators, and is committed in dealing with open challenges. It is important to promote green pharmacy innovation and develop strategies to minimize production-related residues, while also refining methodologies to evaluate the environmental impact of each drug. At the same time, feasible and concrete actions must be defined to limit the environmental impact of healthcare services and medicinal products. In this context, it is also important to acknowledge that the environmental burden of pharmaceuticals extends beyond their residues in ecosystems. The manufacturing processes, distribution chains, packaging materials, and disposal of pharmaceuticals contribute to greenhouse gas emissions and other climate-related impacts throughout the entire life cycle. Integrating life-cycle assessment methodologies with ecotoxicological and prescribing appropriateness frameworks would offer a more holistic understanding of the environmental sustainability of medicines. Monitoring campaigns should also be intensified and better focused on the most urgent medications and the most affected geographical areas.

Citizens also play a fundamental role on the appropriate use and disposal of drugs. It is therefore urgent to start awareness campaigns for patients, caregivers, and healthcare professionals to inform them about the environmental impact of medicines in surface waters. Finally, integrating the concept of planetary health into the education of future generations is essential to promote the culture of One Health. Future research should therefore prioritize the establishment of standardized metrics to assess the environmental impact of medications, evaluate the long-term efficacy and sustainability of intervention and prevention strategies, and enhance interdisciplinary collaboration across all stakeholders. This integrated approach is essential to effectively translate the One Health framework into actionable, evidence-based policies and practical interventions that address the complex interdependencies between human, animal, and environmental health.
